# The role of English as a foreign language learners’ grit and foreign language anxiety in their willingness to communicate: Theoretical perspectives

**DOI:** 10.3389/fpsyg.2022.1002562

**Published:** 2022-09-14

**Authors:** Minqi Wang, Hui Wang, Yan Shi

**Affiliations:** Faculty of Education, Northeast Normal University, Changchun, China

**Keywords:** EFL learners, grit, foreign language anxiety, willingness to communicate, emotional factors

## Abstract

Learners’ willingness to communicate in a foreign language is regarded as a critical issue in educational contexts, so the role of emotional factors in learners’ willingness to communicate has drawn the attention of investigators. This review investigated the studies on the relationship between English as a Foreign Language (EFL) learners’ grit, foreign language anxiety, and willingness to communicate. This review showed a significant relationship between learners’ grit and willingness to communicate. The earlier studies showed that gritty learners with incessant inspiring efforts are more likely to communicate in a foreign language. This review also indicated that lower anxious learners tend to have more willingness to communicate. Earlier studies also indicated that the theories, such as broaden-and-build, positive psychology, dynamic system, affective filter, and attentional control can justify the relationships between these constructs. Moreover, the study has some pedagogical implications and suggestions for teachers, learners, syllabus designers, material developers, teacher educators, policy-makers, and advisors. The ideas can improve their awareness of teachers’ willingness to communicate, grit, and foreign language anxiety in educational environments.

## Introduction

In the past, the aim of teaching English was the mastery of the structure of the language. However, in this age of communication, English seems to be playing a major role, and the purpose of teaching the language has shifted from the mastery of structure to the ability to use the language for communicative purposes. Thus, the communication aspect of teaching English has gained importance. Moreover, the ultimate goal of language learning is currently defined as “authentic communication between persons of different languages and cultural backgrounds” (MacIntyre et al., 2003, p. 559). Since oral communication is a major goal of language instruction in both English-speaking and non-English speaking countries, a renewed focus on the significance of second or foreign language education has developed. Many developing countries have adopted policies that are designed to reform English education in order to enable citizens to achieve greater success in English communication proficiency because English remains by far the dominant world language. A recognizable goal of English as Second Language (ESL) and English as a Foreign Language (EFL) learning is to facilitate better communication and understanding among individuals from different cultural and language backgrounds (Raja et al., 2022).

It is not uncommon to find that some English learners are willing to communicate in English in the classroom, while others are reticent. It is also noticeable that less proficient learners may communicate in English outside the school when they are willing to, while on the contrary, highly proficient learners may be much less likely to talk (Allahyar, 2021). Willingness to communicate, a construct initially developed to account for some of the individual differences in first language communication, has gradually expanded to become an important factor in explaining second language learning and communication. In non-English speaking countries, especially Asian countries, there is an increasingly important need for people to develop English language communicative competence. Despite the uniform challenges in communicative competency, Students’ socialization patterns and their willingness to communicate are not uniform (Lee and Drajati, 2019). According to MacIntyre et al. (1998), willingness to communicate in a foreign language is conceptualized as “a readiness to enter into discourse at a specific time with a specific person or persons’ using a L2” (p. 547), driving some learners to seek opportunities for L2 speaking, and others to avoid communication and remain silent in the classroom (MacIntyre, 2007). Some students are willing to get involved in a variety of activities that require communication, whereas some others are worried about, hesitant, or reluctant to communicate even when the opportunities are readily available (Khajavy et al., 2018).

In past recent years, willingness to communicate has been viewed as a measurement of language program success (Mehrgan, 2013); in other words, a language program success, has been measured according to that program’s ability to augment learners’ willingness to communicate. MacIntyre et al. (1998) argued that in order to have a successful language program, the focus should be on Students’ willingness to communicate. This is a failure if the program is not able to increase learners’ willingness to communicate. Thus, for improving the language teaching situation, the factors influencing willingness to communicate should be investigated and should be recognized.

Yashima et al. (2004) strongly suggested that future research on willingness to communicate in English should focus on contextual or situational variables that make a person more or less willing to communicate both in English classroom settings, and outside classes. That is, it is necessary to look into how negative and positive emotional constructs influence communication behavior and make them more or less willing to communicate. One significant variable that considerably affects language learning is foreign language anxiety (Binti and Rashid, 2018), which is also strongly connected to the willingness to communicate (Kruk, 2022). MacIntyre and Gardner (1994, p. 284) defined foreign language anxiety as “the feeling of tension and apprehension specifically associated with second language contexts, including speaking, listening, and learning.” Studies considered a negative relationship between foreign language anxiety and willingness to communicate (Dewaele, 2019; Lee and Lee, 2020). However, there is not any conceptual review that considers the theoretical relationship between foreign language anxiety, as a negative emotional construct, and willingness to communicate. This review tries to fill the gap by considering theoretical relationships on this issue.

Investigating the effect of positive emotional construct in language learning has drawn the attention of many investigators (Fathi et al., 2021; Greenier et al., 2021; Wang et al., 2022). The significance of positive psychology, as an approach to identifying human characteristics and creating emotional sources to be successful in regular affairs (Wang et al., 2022), has encouraged investigators to seek affective and individual variables of both teachers and learners. Positive psychology is theoretically underpinned by the broaden-and-build theory of positive emotions, explaining that experiencing feelings, such as joy, love, contentment, engagement, resilience, grit, interest, self-efficacy, and emotional regulation “broaden people’s momentary thought-action repertoires and build their enduring personal resources” (Derakhshan et al., 2022, p. 2). Grit, as an emerging non-cognitive positive emotional construct in language learning, is a significant individual variable that was first introduced by Duckworth et al. (2007) in the field of psychology. This personality trait is defined as “one’s passion and perseverance for long-term goals” (Duckworth et al., 2007, p. 1087). Roughly speaking, grit can be counted as having a lasting commitment to one’s goals and unwavering patience in facing failures. Duckworth et al. (2007) defined grit in a broad sense concerning success in the workplace, military, education, and sports. Also, in second and foreign language development, grit can play a crucial role since language acquisition requires extended effort (Hakuta et al., 2000), perseverance, and patience (Alamer, 2021). Recent investigations indicate a positive relationship between learners’ grit and willingness to communicate (Lee and Hsieh, 2019; Lee and Lee, 2020). Despite this vast volume of interest devoted to this issue, there is not any conceptual review to consider the theoretical relationship between learners’ grit and willingness to communicate in a foreign language. Therefore this review tries to inspect the theoretical perspectives of the relationship between grit and willingness to communicate in order to shed light on the significance of positive psychological constructs in language use. Having awareness of the role of positive and negative emotions in the intention of language learners to use language in a different context is useful for teachers and teacher educators to employ effective methods in their classrooms that would improve willingness to communicate in English.

## Literature review

### Reviewing the concepts of willingness to communicate, grit, and foreign language anxiety

The concept of willingness to communicate emerged from the work on communication in a native language in the late 1950s and early 60s in North America, where interpersonal communication is strongly valued. Although people who communicate well are positively evaluated, some people do not communicate much. The difference in communication behaviors was conceptualized as regularly occurring across situations, as determined by certain personality traits (McCroskey, 1997). This concept was called willingness to communicate which, MacIntyre and Charos (1996) pointed out, refers to “a stable predisposition toward communication when free to choose to do so” (p. 7). MacIntyre et al. (1998) defined it as the Students’ willingness to participate in specific interactive situations or to involve in doing educational and communicative tasks. Kruk (2019), in another definition, indicated that willingness to communicate refers to a learner’s state of cognitive planning to apply the target language in his communication. McCroskey and Baer (1985) also defined willingness to communicate from the psychological perspective. They indicated that willingness to communicate refers to “personality orientation which explains why one person will communicate and another will not under identical or virtually identical situational constraints” (p. 3). MacIntyre and Vincze (2017) considered willingness to communicate as the main objective of foreign language learning since the intention to communicate can result in authentic communication behavior, which leads to an increase in foreign language proficiency. Öz et al. (2015) believed that willingness to communicate is indeed a multi-faceted construct that integrates affective, social-psychological, linguistic, and communicative variables and can describe, explain, and predict language learners’ communicative behavior in an L2. All of the learners are not similar in their foreign language willingness to communicate when they have an opportunity to use language and some of the learners do not have enough tendencies to participate in the foreign language classrooms. Therefore, willingness to communicate is regarded to be one of the individual differences variables (Khajavy et al., 2016).

According to MacIntyre et al.’s (1998) model, foreign language learners’ willingness to communicate has been investigated from trait-like and dynamic, and situated dimensions (Dewaele and Dewaele, 2018). The trait-like or psychological dimension of willingness to communicate is correlated with foreign language anxiety (Liu, 2018), self-confidence, and motivation (Lee and Hsieh, 2019). On the other hand, the dynamic and situated dimensions of willingness to communicate refer to the social and contextual features of education, including interlocutors (Toyoda and Yashima, 2021), themes of interaction (Pawlak et al., 2016), instructors (Zarei et al., 2019) and cooperative peers (Khajavy et al., 2016). The concept of willingness to communicate has recently drawn the attention of many investigators.

Studies showed the positive influence of learners’ willingness to communicate on their academic achievement. Khajavy et al. (2016) believed that learners who are more willing to communicate in a foreign language will have better achievement in their learning because by using language communicatively, their learning will be facilitated. Moreover, the study of Mirici and Sari (2021) revealed that learners’ intention to communicate in a foreign language may lead them to involve in educational contexts and interaction, which, in turn, leads to academic success. Learners with higher levels of willingness to communicate are assumed to seek more opportunities in order to involve in authentic educational contexts, which consequently allow them to learn their foreign language (Al-Murtadha, 2021). Some studies have verified the positive relationship between learners’ willingness to communicate and their cognitive capabilities, including speaking (Rahimi and Fathi, 2022) and writing (Gholami and Barzegar, 2018). Tousi and Khalaji (2014) conducted a research study on the concept of willingness to communicate and its impact on the speaking ability of EFL learners and found a significant correlation between Students’ willingness to communicate and their language achievement and a positive relationship between willingness to communicate and learners’ speaking skill.

The direct and indirect influences of the educational environment and positive and negative emotions were studied by Joe et al.’s (2017). They investigated Korean EFL learners’ willingness to communicate and its emotional effects. Their study revealed that FL learners’ willingness to communicate was influenced by their autonomy, competence, relatedness, teacher academic support, teacher emotional support, and classroom mutual respect, and learners’ regulation. They indicated that willingness to communicate is specified by the interaction of learner and social variables. Learners’ willingness to communicate can be significantly influenced by some other positive emotional constructs, such as foreign language self-confidence (Aoyama and Takahashi, 2020; Fatima et al., 2020), motivation (Kruk, 2022), and foreign language enjoyment (Khajavy et al., 2018; Lee, 2020). This review investigates the studies on the relationship between grit, as another positive emotional construct, and willingness to communicate. Moreover, the relationship between foreign language anxiety, as a negative emotional construct, and learners’ willingness to communicate will be contemplated in this study.

Considering grit as another variable in this study, Cross (2014) described an ability to tolerate difficulties though preserving the wish for long-term purposes. Duckworth et al. (2007) defined grit “as a compound trait characterized by perseverance and passion for long-term goals” (p. 1087). They also noted that grit includes “working strenuously toward challenges, maintaining effort and interest over the years despite failure, adversity, and plateaus in progress” (p. 1088). They categorized this higher-order concept into two components: steadiness in interests and persistence in effort despite difficulties (Duckworth et al., 2007). Li and Dewaele (2021) defined steadiness in interests as learners’ ongoing enthusiasm for a long-standing objective regardless of the difficulties, problems, disappointments, dissatisfaction, or hardships they may face. They also maintained that persistence in the effort is related to learners’ predisposition to invest constant efforts on the trail of long-term purposes. Karlen et al. (2019) also highlighted these components in Grit Scale as a measurement of the grit construct. Cohen (2015) underscored the teachability of grit as a dynamic construct, and numerous educational organizations are involved in the promotion of learners’ grit through providing instructional programs.

Some studies have been done on the relationship between learners’ grit and negative emotional constructs such as foreign language anxiety (e.g., Li and Dewaele, 2021; Sudina and Plonsky, 2021), and burnout (Teuber et al., 2021). Some research has affirmed the significant positive correlation between grit and positive emotional constructs such as self-efficacy (e.g., Usher et al., 2019; Alhadabi and Karpinski, 2020), wellbeing (Arya and Lal, 2018), motivation (e.g., Changlek and Palanukulwong, 2015; Teimouri et al., 2020), and academic engagement (Nelson and Baltes, 2019). There are also some investigations showing a positive relationship between grit and test emotions (Datu and Fong, 2018), and self-efficacy (Alhadabi and Karpinski, 2020).

Moreover, anxiety, as a negative emotion, is described as a state of sensitive awareness correlated with an augmentation in stress as a result of ambiguity (Wells and Matthews, 1996). Anxiety can be classified into a trait, state, and situation-specific anxiety (Abrar, 2018). Ellis (1994) defined state anxiety as “the apprehension that is experienced at a particular moment in time as a response to a definite situation” (p. 145). Alshahrani (2016) stated that state anxiety is a provisional affective state that each person may feel when they are exposed to danger in a particular situation. According to Elwood et al. (2012), “trait anxiety is an individual’s tendency to appraise situations as threatening, avoid anxiety-provoking situations, and demonstrate high baseline physiological arousal” (p. 23). Erdiana et al. (2020) indicated that trait anxiety, as a stable one, made people feel nervous in a wide range of situations. Situation-specific anxiety is a type of anxiety that is stimulated by a particular situation, for example, public speaking or participation in class (Ellis, 1994). Horwitz et al. (1986) introduced foreign language anxiety as a situation-specific anxiety. Many different researchers (e.g., Aydin, 2018, Ulupinar, 2018, Russell, 2020; Hu et al., 2021; Toyama and Yamazaki, 2021) have highlighted foreign language anxiety in the field of education. In an educational context, Horwitz et al. (1986) defined foreign language anxiety as “a distinct complex of self-perceptions, feelings, and behaviors related to classroom language learning arising from the uniqueness of the language learning process” (p. 127). Gardner and MacIntyre (1993) pointed out that “foreign language anxiety is fear or apprehension occurring when a learner is expected to perform in the second or foreign language” (p. 59). They linked it to the stimulation of the autonomic nervous system. Horwitz et al. (1986) categorized foreign language anxiety construct into (a) test anxiety, (b) fear of negative evaluation, and (c) communication apprehension. Cakici (2016) mentioned that test anxiety is regarded as learners’ fear of experiencing failure in academic evaluation in testing contexts. He also stated that negative evaluation is concerned with worrying about individuals’ negative judgments. Communication apprehension, based on Horwitz et al.’s (1986) definition, refers to “a distinct complex of self-perceptions, beliefs, feelings, and behavior related to classroom language learning arising from the uniqueness of the language-learning process” (p. 28). Communication apprehension is a critical element in limiting the received comprehensible input, and it plays a vital role in determining accomplishment in educational contexts (Darmawangsa et al., 2020). Foreign language anxiety as a component of a situation-specific anxiety has been reviewed in this conceptual analysis.

Studies have shown several sources of foreign language anxiety such as minor levels of self-confidence (Tridinanti, 2018), lower levels of self-efficacy (Bensalem, 2018), low levels of grit (Liu and Wang, 2021), lack of practice (Bárkányi, 2018), low levels of language proficiency (Teimouri et al., 2019), low levels of emotional intelligence (Chen et al., 2021), fear of making mistakes (Suparlan, 2021), insufficient input flooding, first language overuse, cultural background factors (Shan et al., 2020), socio-economic status (Ali et al., 2021), and teachers’ negative impression about learners’ academic performance (Liu and Wu, 2021).

Learners’ cognitive performances have been affected by their foreign language anxiety. Mede and Karaırmak (2017) pointed out that “foreign language anxiety significantly affects the domains of language achievement, learners’ actual proficiency and performance, gender, prior foreign language experience, negative evaluation, and self-evaluation” (p. 119). Foreign language anxiety has been widely considered a leading factor in academic achievement and language proficiency (MacIntyre, 2017). Studies have testified that higher levels of foreign language anxiety are negatively correlated with foreign language proficiency, and with positive orientation and peer emotional support (Zheng and Cheng, 2018). It is worth noting that foreign language anxiety is negatively affected by input, processing and output as the phases of cognitive processing (MacIntyre and Gardner, 1994). Regarding the relationship between foreign language anxiety as a negative type of emotion and cognitive skills, Fallah and Movahed (2014) indicated that there was a negative correlation between foreign language anxiety, and listening, reading, speaking, and writing skills, and learners’ academic achievements were negatively influenced by anxiety. Hu et al. (2021) also found that learners’ foreign language anxiety was negatively associated with their foreign language skills.

### Discussing the role of learners’ grit in willingness to communicate in foreign language

This review used broad-and-build theory, positive psychology, and dynamic system theory as theoretical frameworks, to explicate the relationship between positive emotional construct, like grit, and learners’ willingness to communicate. According to broad-and-build theory, gritty language is more engaged in language learning (MacIntyre and Gregersen, 2012). According to Fredrickson (2013), positive emotional states comprise a vital issue for broadening people’s temporary thought–action and attention capacities, building their individual resources for the future, enhancing their resilience in adverse situations, and developing their creativity in relation to problem-solving. According to Fredrickson’s (2001) broad-and-build theory, a positive classroom environment where the teacher is friendly and supportive helps increase Students’ interest in exploring opportunities to communicate in the target language. Using this theory, MacIntyre and Gregersen (2012) asserted that learners’ positive emotional states can contribute to overcoming their negative emotions when learning/using a foreign language. They also argued that positive emotions can boost the ability of learners to learn and process foreign languages, and they contended that enjoyment expedites the growth of learners’ thought-action repertoire to be proficient in language learning, and contribute increasing language knowledge. strong desire, positive attitude to learn, and long-term commitment to learn the target language. Lee and Hsieh (2019) studied the relationship between Taiwanese EFL learners’ grit, foreign language self-confidence, and willingness to communicate. Their study indicated that grit acts as a significant predictor of learners’ foreign language willingness to communicate. They also argued that Taiwanese EFL students who attempt continuously to attain their objectives (i.e., becoming a proficient English speaker) try to look for more chances to develop their communicative skills in English educational contexts. Fathi et al. (2021) used a structural equation modeling approach in order to study the relationship between grit, foreign language anxiety, and willingness to communicate. Having used broad and band theory, they found a significant role of grit in predicting learners’ willingness to communicate. They argued that EFL learners, with higher levels of grittiness, have higher extents of perseverance of effort, which may induce industry among learners to undertake demanding tasks and give precedence to the communicative and efficient tasks. They also argued that learners, with higher levels of grittiness, tend to communicate in English with their peers and educators to boost their language competence. They maintained that gritty EFL learners have higher levels of achievement-oriented features, which inspire them to do their best to achieve their educational objectives in English language learning contexts. Wang et al. (2021) used the broaden-and-build theory of positive emotions to explicate the effect of learners’ positive emotional states on foreign language learning and use. They argued that hat argued that positive emotions act as unique variables in foreign language learning. Positive emotional states can extend learners’ attentional span, allowing them to retain data for learning, which increases the problem-solving ability among learners to use language appropriately.

Positive psychology as another theoretical framework can also justify the relationship between grit and willingness to communicate. Seligman and Csikszentmihalyi’s (2000) positive psychology is regarded as a theory that can relate learner grit to linguistic skills. Traditionally, psychologists have highlighted the deficiencies and negative emotions among learners and teachers, and they have made an effort to decrease them (Dewaele and Alfawzan, 2018; Derakhshan et al., 2021). Positive psychology, as a modern approach to learning a foreign language, has been expanded in recent years (Wang et al., 2021). It tries to illuminate the optimal educational situations and processes for the achievement of learners and teachers (Jiang, 2020). Fathi et al. (2021) also mentioned that positive psychological constructs can facilitate a person’s achievement in language learning and use. On the other hand, Abdolrezapour (2018) stated that positive emotions tend to widen learners’ viewpoints and open their viewpoint to absorb the language. Fredrickson and Cohn (2008) stated that positive affect is the major trigger of active engagement with the learners’ environment and his/her will to participate in classroom activities.

Dynamic system theory can also relate the interaction of different variables to a willingness to communicate. As a novel approach to grit, Lee (2020) divided the construct of grit into the perseverance of effort and consistency of interests to investigate the role of each sub-component in Korean middle school learners’ willingness to communicate. Using hierarchical regression analyses, his study revealed that perseverance of effort is significantly correlated with willingness to communicate in a foreign language, while the consistency of interests is not significantly correlated with willingness to communicate in a foreign language among all learners. His study suggested that L2 teachers, who instruct in a relatively monolingual and monocultural EFL classroom, can boost learners’ willingness to communicate by encouraging continuous efforts to initiate English communication. He justified his results from grit and socio-educational points of view. He argued that EFL learners with higher levels of grittiness are likely to function diligently toward carrying out demanding oral tasks, therefore they tend to initiate English communication in educational contexts. He also contended that Korea’s social context is likely to enhance EFL secondary and university learners’ foreign language willingness to communicate. Cheng (2021), in his study, found that the persistence of the effort dimension of grit, compared to its counterpart, the consistency of interest, exhibited a superior predictive power over learners’ intention to communicate in a foreign language. He argued that how consistent one displays focused interest in specific language learning over time is less relevant to motivating learning outcomes than how unflaggingly one shows dedication and engagement in language tasks even in the face of difficulty. He also found that L2 self-mindsets, including ideal L2 self/own, ideal L2 self/other, and ought to L2 self/own, as well as, grit, are the significant contributors to learners’ willingness to communicate. Lan et al. (2021) investigated the emotional processes involved in educational contexts in the relationship between the ideal L2 self and foreign language willingness to communicate. They found the mediational role of grit and shyness in this correlation. They argued that the ideal L2 self is considered an antecedent of grit. They declared that language learners who have a clearer vision of their future ideal L2 self are inclined to represent their grittiness in following their long-term educational objectives and achieve their desired image of self as a foreign language learner.

It can be argued that there is a reciprocal relationship between grit and willingness to communicate through the mediation of motivation. While motivation and emotions are conceptually distinct, they are nonetheless inextricably strongly linked. The association between these concepts appears to be bidirectional and deeply multi-faceted. Lee and Hsieh (2019) asserted that motivation had the largest direct positive influence on willingness to communicate in a foreign language, establishing that learners’ willingness to communicate in English is mostly directly accounted for by their motivation to learn the language. Interestingly, the role that motivation plays is two-dimensional, as it also contributes to the willingness to communicate in a foreign language indirectly through encouraging positive emotions among learners (Lee and Hsieh, 2019).

### Discussing the role of learners’ foreign language anxiety in willingness to communicate in foreign language

In order to justify the relationship between learners’ anxiety and willingness to communicate in a foreign language, dynamic systems theory, Krashen’s (1982) affective filter theory, and Eysenck et al.’s (2007) attentional control theory were used as theoretical frameworks in this review. Concerning the relationship between learners’ foreign language anxiety and willingness to communicate, Zhou et al. (2020) used dynamic systems theory and confirmed the interactive effect of foreign language competence and foreign language anxiety on willingness to communicate in the Chinese context. They argued that foreign language anxiety may impair foreign language learners’ self-assessment of their own language competence, which may, in turn, lead to less willingness to initiate communication in the target language. Horwitz (2017) also found out that foreign language anxiety has a negative significant correlation with the willingness to communicate. Ehsani and Moghaddam (2021) found a negative correlation between foreign language anxiety and willingness to communicate. They argued that less anxious learners are inclined to have higher levels of intention to communicate. Fujii (2021) looked into the correlations between foreign language anxiety and willingness to communicate, the differences in willingness to communicate between high-anxious and low-anxious learners, and learners’ intention to communicate effectively in educational contexts. His study found a significant, negative relationship between foreign language anxiety and willingness to communicate, and the differences between high-anxious and low-anxious learners in terms of willingness to communicate were significant. His study also emphasized the role of educational activities in the correlation between willingness to communicate and foreign language anxiety. Based on his study, learners, with higher levels of willingness to communicate in meaning-focused activities are more inclined to have lower anxiety levels. Dewaele (2019), in their study, found that adult foreign language Spanish learners’ willingness to communicate was reduced by foreign language anxiety but reinforced by enjoyment. They also argued that learners with lower levels of foreign language anxiety are not a warranty for an amplified willingness to communicate among learners, since disengaged learners normally lack enjoyment as well as anxiety, whereas involved learners may be exposed to higher levels of foreign language anxiety and foreign language enjoyment instantaneously.

Dewaele and Pavelescu (2019), in a case study, also investigated two Romanian high school learners’ foreign Language enjoyment, foreign language anxiety, and willingness to communicate. They used dynamic system theory in order to justify their approach. Their study indicated that willingness to communicate was correlated with foreign language enjoyment and foreign language anxiety in dynamic and idiosyncratic ways. They argued that anxiety is a component of a dynamic system and interacts constantly with numerous learner and contextual variables, such as language proficiency, self-related appraisals, interpersonal relationships, and conversation topics, which can affect learners’ willingness to communicate as a dynamically evolved construct in interaction.

Other theoretical frameworks can also validate the relationship between learners’ foreign language anxiety and L2 willingness to communicate. For instance, Krashen’s (1982) affective filter theory can also justify this negative correlation. Wang and Wu (2020) indicated that when learners’ affective filters are high, the created mental block makes it impossible for them to acquire or be comfortable with the input. Thus, the affective filter level will create a hostile environment in which the students feel insecure about themselves, and their capacities to use the language to communicate. Using Krashen’s (1982) affective filter theory, García Uquillas (2021) argues that anxiety may hinder input from transforming into intake, i.e., transforming uttered elements into comprehension. He further postulates that the more successful methods are the ones that encourage a low filter by creating a relaxed atmosphere in which students do not feel defensive. He mentioned that instructors should lower the affective filter since all factors involved in the affective filter, such as higher anxiety, lower motivation, and lower self-efficacy may bring a negative influence on language production. That is, the affective filter plays an important role in the foreign language learning process. Nath et al. (2017) asserted that some learners had a high filter which had negative consequences on their oral production of the target language, whereas other learners had a low filter that encouraged the appropriate oral production of the language and use of the metacognitive strategies for their own sake.

Wells and Matthews (1996) mentioned that foreign language anxiety can be characterized by inadequate control over disturbing opinions and attentional and cognitive predispositions, leading to an excessive concentration on undesirable incentives. Zhou et al. (2020) mentioned that Eysenck et al. (2007) attentional control theory can justify the reason behind the negative correlation between foreign language anxiety and cognitive processing performance. They maintained that foreign language anxiety limits the attentional control of learners in adverse situations such that learners are more prone to threat-related stimuli or distractors.

## Implications and further suggestions

This review examined the role of EFL learners’ grit and foreign language anxiety in their intention to communicate. Earlier studies hint that gritty learners tend to have higher levels of willingness to communicate. Moreover, studies showed that less anxious learners are more likely to communicate in a foreign language. This review includes some pedagogical implications for teachers, learners, syllabus designers, teacher educators, educational policy-makers, and advisors. Also, different associations and related authorities can benefit from this review. For instance, a better understanding of Students’ willingness to communicate in the target language may help language teachers improve the communicative language teaching approach and curriculum design to provide more communication opportunities for language learners, more importantly, encourage actual engagement in communication behaviors, and finally, facilitate second/foreign language learning and acquisition. More specifically, language instructors can enhance the level of Students’ willingness to communicate through the following ways: raising Students’ opportunity to talk by reducing the amount of teacher talk and allowing adequate wait-time; letting students produce language without restrictions, uncontrolled use of language; take responsibility to engage all students evenly and equally in classroom activities; videotaping themselves in the classroom, reflect on their interactional behavior to see if it has extended or limited the opportunity for the students to enter dialogues; increasing their own awareness of what interaction strategies work or do not work with specific students, and giving the instruction that lends itself to more giving and receiving of unpredictable information. Also, the EFL students can receive different strokes by doing well or never doing well. Therefore, strokes might distinguish between successful and unsuccessful learners.

One of the most important implications is that by lowering language anxiety, Student’s willingness to communicate may increase and as a result, lead to more target language use in the classroom or even out of the classroom. Thus, in order to encourage Students’ willingness to communicate and enhance their involvement in the class activities teachers need to reduce Students’ FL anxiety in a class by making the class atmosphere more friendly and comfortable. In a less threatening atmosphere, learners’ anxiety may reduce while their level of perceived competence increases. Therefore, teachers need to make a relaxed and intimate atmosphere to increase the willingness to communicate among students and as a result, enhance language learning in the class. Using activities like role play, games, small group work, pair work, small group discussion, and presenting the lecture may make the class atmosphere less threatening for students. EFL teachers can also mitigate learners’ anxiety level by (a) raising awareness that errors are signs of learning and changing their negative attitudes toward mistakes, (b) correcting them unobtrusively when it is necessary, and (c) praising them for their correct utterances (i.e., emotional support). Teachers should make efforts to encourage students not to feel embarrassed after they made mistakes. Teachers should also regulate learner emotions by adopting a nuanced emotion-regulation approach that focuses on promoting learners’ positive emotions and affect and reducing the lingering effects of negative emotions. According to MacIntyre and Wang (2021), utilizing mixed positive and negative emotions regulation activities can more effectively develop learners’ willingness to communicate in foreign language.

Another implication of this review is to develop learners’ grit to enhance their willingness to communicate. Foreign language teachers, who instruct in a relatively monolingual and monocultural EFL classroom, can boost learners’ willingness to communicate in the foreign language by providing affective and capacity supports, as well as creating positive classroom environments. It is believed that instructing learners on techniques to improve their grit should be given the same priority as other language skills in the EFL context. EFL teachers are thus suggested to increase their learners’ grit by making it a slogan in their classrooms and allocating more long-term activities and tasks. For example, EFL teachers can have a positive rapport with their students to help them identify the fact that their continuing language learning purposes are decisive and therefore ask them to set some feasible, detailed, and definite long-term purposes for their language learning with a realistic time frame in mind. One valuable strategy to instruct EFL students to differentiate and deal with numerous difficulties in the courses of their language learning is likely to use the method of “wish, outcome, obstacle and plan” (Oettingen, 2014). One more operative grit-building strategy is integrating more learner reflection with different tasks, accomplishments, or assessments. Moreover, EFL educators are required to preserve high prospects over numerous instructive and communicative contexts and inspire their students to persist and preserve.

Syllabus designers should devote special effort to developing grit in learners. When syllabus designers develop material for EFL classes, they should consider each learner’s ability to persevere, practice, and his or her desired goal and interest. In this regard, Christensen and Knezek (2014) referred to the point that putting more emphasis on non-cognitive characteristics like grit may play an important role in supporting learner success in academic and non-academic settings. Syllabus designers should design a sequence of activities and practices in order to encourage students to engage actively in-class activities. They can develop strategies for designing curriculum reform based on positive psychology.

Furthermore, EFL learners should be informed of the significance of willingness to communicate in language learning in general and the effects of both foreign language anxiety and grit, in learning a target language, in particular. English major University students with the help of their teachers can find out some way to overcome their anxiety in classes to improve their self-confidence to achieve their learning goals. Moreover, teacher training courses can reach their ideal goals by considering the importance of grit and willingness to communicate. They can implement some instructional or cognitive changes in a large population of students or teachers, by providing teachers and teaching them how to increase their grit and necessary strategies based on transactional analysis. Educational policy-makers should hire experienced teachers, as the instructive experience can be an important issue for increasing grit and willingness to communicate among learners. They can increase learner grit and decrease learner foreign language anxiety by holding academic workshops that offer teachers some strategies. They can ask teachers to do their best within varied educational contexts. They must build up teaching effectiveness by providing contexts for observations of other teachers’ activities and mastery experiences to decrease foreign language anxiety in particular ranges of the instruction. They should also provide critical thinking, creativeness, and motivation into the education in classrooms, which encourages grit and willingness to communicate. The importance of grit, anxiety, and willingness to communicate can motivate advisors to expand their horizons to identify learners’ sources of foreign language anxiety, grit, and to probe the reasons for willingness to communicate.

Many cross-sectional studies have been done on the role of emotional constructs in learners’ willingness to communicate. A longitudinal qualitative study is necessary to examine the effect of emotional constructs on learners’ willingness to communicate in English in various situations both inside and outside class. It would also prove fruitful to examine learners’ willingness to communicate in English through a variety of data collection methods, such as classroom observations, interviews, and the responses from communication partners (teachers or peers) to examine the issue in-depth in a specific situation. Instructor’s perceptions of the communication behaviors of their learners are also worth investigating. In order to increase learners’ willingness to communicate in English, teachers need to monitor various aspects of teaching styles and classroom management, which begs further exploration. Future research can be conducted about the relationship between teaching style and Students’ willingness to factors in a certain situation to enhance willingness to communicate is a big issue to explore. Moreover, the relationship between willingness to communicate and other positive psychological constructs, such as academic engagement, wellbeing, foreign language enjoyment, self-efficacy, pedagogical love, and resilience can be investigated in the future. Moreover, future predictive models of willingness to communicate in a foreign language can based on grit and anxiety be expanded based on dynamic complex system theory. Factors that could be more closely examined include the relationship between age, gender, background, personality, intercultural communication experiences, and learners’ willingness to communicate.

## Author contributions

All authors listed have made a substantial, direct, and intellectual contribution to the work, and approved it for publication.
